# Effect of silver nanospheres and nanowires on human airway smooth muscle cells: role of sulfidation†

**DOI:** 10.1039/d0na00745e

**Published:** 2020-10-09

**Authors:** Charalambos Michaeloudes, Joanna Seiffert, Shu Chen, Pakatip Ruenraroengsak, Leo Bey, Ioannis G. Theodorou, Mary Ryan, Xiaoxing Cui, Jim Zhang, Milo Shaffer, Terry Tetley, Alexandra E. Porter, Kian Fan Chung

**Affiliations:** National Heart & Lung Institute, Imperial College London Dovehouse St London SW3 6LY UK f.chung@imperial.ac.uk; Department of Materials, London Centre for Nanotechnology, Imperial College London SW3 UK; Faculty of Pharmacy, Mahidol University Bangkok 10400 Thailand; Faculty of Medicine, University of Malaya Kuala Lumpur 50603 Malaysia; Nicholas School of Environment, Duke Global Health Institute, Duke University Durham USA

## Abstract

*Background*: The toxicity of inhaled silver nanoparticles on contractile and pro-inflammatory airway smooth muscle cells (ASMCs) that control airway calibre is unknown. We explored the oxidative activities and sulfidation processes of the toxic-inflammatory response. *Method*: Silver nanospheres (AgNSs) of 20 nm and 50 nm diameter and silver nanowires (AgNWs), short S-AgNWs, 1.5 μm and long L-AgNWs, 10 μm, both 72 nm in diameter were manufactured. We measured their effects on cell proliferation, mitochondrial reactive oxygen species (ROS) release and membrane potential, and also performed electron microscopic studies. *Main results and findings*: The greatest effects were observed for the smallest particles with the highest specific surface area and greatest solubility that were avidly internalised. ASMCs exposed to 20 nm AgNSs (25 μg mL^−1^) for 72 hours exhibited a significant decrease in DNA incorporation (−72.4%; *p* < 0.05), whereas neither the 50 nm AgNSs nor the s-AgNWs altered DNA synthesis or viability. There was a small reduction in ASMC proliferation for the smaller AgNS, although Ag^+^ at 25 μL mL^−1^ reduced DNA synthesis by 93.3% (*p* < 0.001). Mitochondrial potential was reduced by both Ag^+^ (25 μg mL^−1^) by 47.1% and 20 nm Ag NSs (25 μg mL^−1^) by 40.1% (*both at *p* < 0.05), but was not affected by 50 nm AgNSs and the AgNWs. None of the samples showed a change in ROS toxicity. However, malondialdehyde release, associated with greater total ROS, was observed for all AgNPs, to an extent following the geometric size (20 nm AgNS: 213%, *p* < 0.01; 50 nm AgNS: 179.5%, *p* < 0.01 and L-AgNWs by 156.2%, *p* < 0.05). The antioxidant, *N*-acetylcysteine, prevented the reduction in mitochondrial potential caused by 20 nm AgNSs. The smaller nanostructures were internalised and dissolved within the ASMCs with the formation of non-reactive silver sulphide (Ag_2_S) on their surface, but with very little uptake of L-AgNWs. When ASMCs were incubated with H_2_S-producing enzyme inhibitors, the spatial extent of Ag_2_S formation was much greater. *Conclusion*: The intracellular toxicity of AgNPs in ASMCs is determined by the solubility of Ag^+^ released and the sulfidation process, effects related to particle size and geometry. Passivation through sulfidation driven by biogenic H_2_S can outcompete dissolution, thus reducing the toxicity of the smaller intracellular Ag nanostructures.

## Abstract 2

Sulfidation by biogenic H_2_S outcompetes dissolution of silver nanostructures, thus reducing their toxicity in airway smooth muscle cells.

## Graphical abstract

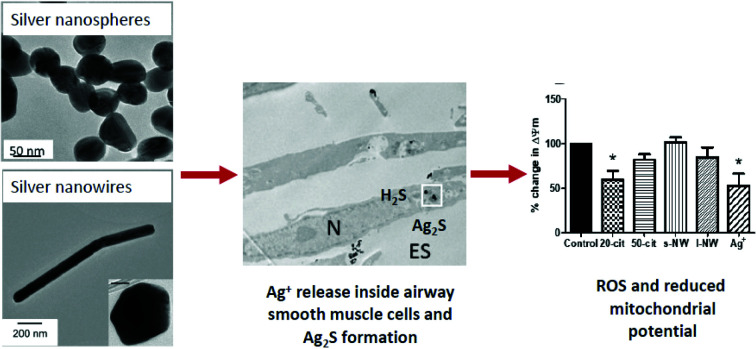

## Introduction

Silver nanoparticles (AgNPs), including silver nanospheres (AgNSs) and nanowires (AgNWs), are increasingly used in a wide range of consumer products including odour-resistant clothing, medical devices, food storage items, cleaning sprays and personal hygiene products.^1^ They are also used in the production of flexible and conductive thin films for flat and touch screens because of their robust electrical conductivity. Inhalation exposure to AgNPs represents a potential occupational risk not only for workers in the nanoparticle manufacturing industry, but also for consumers of products containing these AgNPs. There are reports that up to 14% of consumer products containing AgNPs may release these nanoparticles into ambient air from where they can be inhaled into the lungs.^2,3^

*In vivo* and *in vitro* studies of the effects of AgNPs, particularly AgNSs, have identified cytotoxic and genotoxic effects in airway cells, including airway fibroblasts and epithelial cell lines.^4–6^ Exposure of rats and mice to inhalation or instillation of AgNPs, mainly AgNSs, has been shown to cause lung inflammation, impair lung function and induce bronchial hyperresponsiveness,^7–10^ indicating that airway smooth muscle (ASM) function might be impaired. However, the mechanisms of toxicity of AgNPs, including both AgNSs and AgNWs, on cells in the airways are currently unclear. Dissolution of AgNPs to release silver (Ag^+^) ions may be important^11–13^ because these cations may interact within the mitochondrial leaflet to cause disruption of ATP production, or proton leakage.^4,14–16^ Ag^+^ may also undergo redox reactions with intracellular oxygen, leading to reactive oxygen species (ROS) release and DNA and membrane damage.^17,18^ In addition, the biological reactivity of AgNPs may also arise from their nanoparticle properties, such as their highly reactive surface with activation of reactive oxygen species (ROS).^19,20^ On the other hand, the transformation of Ag^+^ to the highly insoluble Ag_2_S can deactivate reactive silver species in the lungs, as has been shown with AgNSs and AgNWs in alveolar type 1 epithelial cells.^21^

The ASM cell is an important cell in the airways, present from the trachea down to the conducting bronchioles deep in the lungs, where it regulates the luminal diameter, therefore controlling airflow through the airway system. Indeed, in many respiratory diseases such as asthma, the ASM is hypercontractile being sensitive to agents that can constrict the airway smooth muscle, such that the airways can completely narrow and ultimately close.^22^ In addition to being a contractile cell, the ASM cell can also produce pro-inflammatory cytokines and chemokines in response to various external stimuli, and is responsive to growth factors such as TGFβ or PDGF that can increase its proliferation rate.^23,24^ Given the anatomical position of the ASM in the airway wall, nanoparticles inhaled *via* the airways might interact with these cells. However, very little is known about the interaction of these AgNPs with ASM cells. There has only been one study using 110 nm size AgNSs that has reported apoptotic and anti-proliferative effects of these particles on rat ASM cells through the activation of a muscarinic receptor.^25^

We have previously studied the influence of the aspect-ratio on lung inflammation induced by instillation of PVP capped AgNWs and found that longer AgNWs (10 μm long) caused a greater and longer-lasting degree of lung inflammation with a more diverse cytokine production, and induced bronchial hyperresponsiveness compared to shorter AgNWs (1 μm long).^26^ We also showed that, with these AgNWs, there was inactivation of silver after precipitation of crystalline Ag_2_S with limited free Ag^+^ release.

In the present study, we compared the effects of AgNSs with two diameters (20 and 50 nm) and AgNWs with two different lengths, “short” (S-AgNWs; 1.5 μm length) and “long” AgNWs (L-AgNWs; 10 μm length), to those of an ionic silver (Ag^+^) control, on primary human ASM cells *in vitro*. Specifically, we evaluated cellular and mitochondrial cytotoxicity, oxidative stress, DNA damage, and cytokine release, whilst concomitantly examining the uptake and dissolution of these nanoparticles within ASM cells. We also explored the role played by oxidative processes and sulfidation in the toxic-inflammatory response that we have previously reported in other cell types.^21,27^ We explore the hypothesis that the AgNP geometry controls both uptake and dissolution/passivation rates, thus determining the extent of cytotoxic effects on ASM cells.

## Methods

### Synthesis and characterization of AgNPs

All the nanoparticles and nanowires were manufactured and characterised in-house. Detailed synthesis protocols and extensive characterisation of the 20 nm and 50 nm citrate-coated AgNSs (Ag20 and Ag50 citrate) and AgNWs have been described in our previous reports.^21,28–30^ Briefly, Ag20 citrate was synthesized by chemical bath reduction using sodium borohydride (NaBH_4_) (Fisher Scientific, UK) as the reductant and trisodium citrate (Na_3_C_6_H_5_O_7_) (Fisher Scientific, UK) as the stabilizer. For the synthesis of Ag50 citrate *via* chemical bath reduction, trisodium citrate served a dual role as both a reductant and stabilizer. AgNWs of two different lengths: S-AgNWs 72 ± 36 nm in diameter and 1.5 ± 1.4 μm in length and L-AgNWs 72 ± 36 nm in diameter and 10.5 ± 8.5 μm in length, were prepared *via* a modified polyol pathway through the reduction of AgNO_3_ with ethylene glycol (EG, Sigma-Aldrich, UK) in the presence of polyvinyl pyrrolidone (PVP, *M*_w_ ≈ 360k, Sigma-Aldrich, UK), as described by Theodorou *et al.*^31^ The length of the AgNWs was modified by controlling the molar ratio of PVP to AgNO_3_.

All AgNP types were washed by repeated cycles of centrifugation at 10 000*g* with either acetone or ethanol and at least 3 times with de-ionized (DI) water (Millipore Milli-Q gradient system, >18.2 MΩ), dispersed in DI water in sealed glass vials and stored at 4 °C in the dark. The purity of the as-synthesised particles was confirmed with energy dispersive X-ray spectroscopy (EDS) to ensure that sulfidation of AgNPs had not occurred adventitiously, due to background pollution, and impurities, such as Na^+^ and Cl^−^, were removed by the washing process.^21^ As an ionic control, we used AgNO_3_ with the same concentration of silver as present in the particles (25 μg mL^−1^). For the BrdU assay and assays of cell viability, we also used AgNO_3_ with an ultra-low ionic silver concentration, at ∼1% of the ionic content of silver in 25 μg mL^−1^ AgNPs to represent the very low/negligible dissolution (<1 ppb) measured for AgNSs and AgNWs in DI water and DMEM in our previous studies.^21,28^

### ASM cell culture and AgNP exposure

ASM cells were dissected from the bronchi and trachea from transplant donor lungs, as previously described.^32^ Briefly, ASM cells were suspended in Dulbecco's modified Eagle's medium (DMEM) supplemented with 4 mM l-glutamine, 20 U L^−1^ penicillin–streptomycin, 20 μg mL^−1^ streptomycin, 2.5 μg mL^−1^ amphotericin B and 10% fetal bovine serum (FBS) and then cultured in flasks at 37 °C with 5% CO_2_ in a humidified atmosphere. All the experiments except that using confocal microscopy were performed on cell suspensions for 24 or 72 hours; then supernatants were removed and the cells were washed three times with warm PBS before further experimentation. MTS reagent consisting of alkylthiosulfonates was added to the exposed cells as described in the manufacturer's instructions and the formation of formazan salt was measured at 490 nm using a spectrophotometer. Cell proliferation was quantified using the DNA bromodeoxyuridine (BrdU) assay [Roche Diagnostics, Burgess Hill, UK], following the manufacturer's instructions. Incorporated BrdU was detected by using a peroxidase-labeled anti-BrdU antibody on the fixed cells and then measuring luminescence using a Fluorostar plate reader (BMG, Offenburg, Germany). BrdU is a measure of the DNA synthesis rate, *i.e.* cells with faster proliferation give higher reading compared to those with slower proliferation.

### Malondialdehyde measurements

Free malondialdehyde (MDA) concentrations in the supernatants were measured using a HPLC system with fluorescence detection (Waters, Milford, MA, USA) set at 532 nm for the excitation wavelength and 553 nm for the emission wavelength.^33^ The mobile phase was composed of 40% methanol and 60% water containing 50 mM KH_2_PO_4_ (pH = 6.8) for a Nova-Pak C18 column (Waters, Milford, MA, USA). The detection limit, extraction recovery and analytical precision were 1.8 nM, 75.9%, and 2.2%, respectively.

### Mitochondrial ROS

Mitochondrial ROS was measured using Mitosox™ Red (Invitrogen), a redox sensitive dye which is selectively targeted to the mitochondria and emits red fluorescence after oxidation by superoxide ions. ASM cells were washed with HBSS after AgNP exposure and incubated with 5 μM of MitoSOX reagent for 390 minutes. Red fluorescence was measured at 510/580 by flow-cytometry on a BD FACSCanto™II flo-cytometer [BD Biosciences, UK] analysed using FloJo software.

### Mitochondrial membrane potential (Δ*Ψ*_m_)

Δ*Ψ*_m_ was measured in ASM cells using the cationic dye 5,5′,6,6′-tetrachloro-1,1′,3,3′-tetraethylbenzimidazolylcarbocyanine iodide (JC-1; Invitrogen) which changes its emission from green to red fluorescence on entry into cells. Cells were washed three times with HBSS and incubated with the JC-1 reagent for 30 minutes. Fluorescence was detected on a plate reader (Synergy HT Biotek, Winooski, VT, USA). The Δ*Ψ*_m_ was measured as the ratio between red and green fluorescence.

### TEM and STEM-HAADF/EDX analysis

Serum-starved ASM cells were incubated with SF media or SF media containing dl-propargylglycine (PAG) to inhibit CSE and *O*-(carboxymethyl)-hydroxylamine and hemihydrochloride (CHH) to inhibit CBS, for 1 hour prior to the exposure to the AgNPs for 72 hours. Cells were rinsed briefly in saline (0.9% NaCl) to remove any non-ingested particles and were then fixed in 2.5% glutaraldehyde in 0.1 M PIPES buffer, pH 7.2 for 1 h at 4 °C. The fixatives were then removed by washing cells with 0.1 M PIPES buffer 3 times. Cells were scraped and transferred into 1.5 mL Eppendorf tubes and cell pellets were obtained by centrifugation at 1000*g* for 20 min. The samples were embedded without bulk staining with osmium tetroxide to avoid any dissolution of the Ag nanomaterials during staining. The samples were then dehydrated in a graded ethanol series of 50%, 70%, 95%, and 100% (volume ratio of ethanol to DI-H_2_O) for 5 min each and then rinsed three times in acetronitrile (Sigma) for additional 10 min each, all at room temperature. After dehydration, the samples were progressively infiltrated with a quetol-based resin, created by combining 8.75 g quetol, 13.75 g nonenyl succinic anhydride, 2.5 g methyl acid anhydride, and 0.62 g benzyl dimethylamine. The samples were infiltrated in 50% resin/acetonitrile solution for 2 h, followed by infiltration in a 75% resin/acetonitrile solution overnight and finally in 100% resin for 4 days with fresh resin replaced daily. The embedded samples were cured at 60 °C for 24 h. Thin sections (70 nm) were cut directly into a water bath using an ultra-microtome with a diamond knife with a wedge at an angle of 35°. The sections were immediately collected on bare, 300 mesh copper TEM grids (Agar Scientific), dried and kept under vacuum for TEM analysis. Bright-field TEM imaging was performed on a JEOL 2000 microscope operated at 80 kV using an objective aperture. Multiple cells (>100 per sample) from three exposure experiments culture were viewed using an FEI Titan 80-300 scanning/transmission electron microscope (S/TEM) operated at 80 kV, fitted with a Cs (image) corrector and SiLi EDX spectrometer (EDAX, Leicester UK). HRTEM and STEM-HAADF/EDX analyses were carried out on unstained samples. Over 100 intracellular AgNPs were analyzed for each cell exposure. STEM experiments were performed with a convergence semi-angle of 14 mrad and inner and outer HAADF collection angles of 49 and 239 mrad, respectively. The probe diameter was <0.5 nm.

We examined the contribution of H_2_S enzymes by incubating the ASM cells with an inhibitor of cystathionine γ-lyase (CSE), propargylglycine (3 mmol L^−1^) and an inhibitor of cystathionine β-synthetase (CBS), *O*-carboxymethyl-hydroxylamine hemihydrochloride [CHH] (3 mmol L^−1^).^34,35^

### Expression of CBS, CSE and MPST enzymes

To assess the expression of the H_2_S-producing enzymes, cystathionine β-synthase (CBS), cystathionine γ-lyase (CSE) and 3-mercaptopyruvate sulfurtransferase (MPST) in human ASMCs, cells were seeded on glass cover slips in 24 well-plates and were exposed to 25 μg mL^−1^ AgNPs, S-AgNWs and L-AgNWs for 24 h. Following 24 h of exposure, the cells were washed with Dulbecco's phosphate buffered saline (PBS) and fixed with ice cold methanol for 3 minutes. The cells were blocked with 1% BSA in PBS, pH 7.4 for 30 minutes at room temperature and incubated with primary antibodies to CSE (1 : 200, Santa Cruz Biotechnology, Texas, USA), CBS (1 : 200, Santa Cruz) and 3-MPST (1 : 250, Santa Cruz), the main H_2_S producing enzymes in ASM cells.^35^ Following washing with PBS, cells were incubated with the fluorescently labelled secondary antibodies diluted with a blocking solution (1 : 200 dilution) for 1 h at room temperature. Following further washings, the nuclei were counterstained with Hoechst 33342 (blue color). The glass cover slips were mounted onto the microscope slides with SlowFade® antifade reagent and visualized using a Leica SP5 inverted confocal microscope (Leica, Germany). Cells (*n* = 20 cells) were observed for each sample and three separate experiments were performed with a total number of 60 observed cells. The mean fluorescence intensity of CBS, CSE and MPST was measured using Image J software and the data were presented as mean ± SD.

### Data analysis

The results were analysed using the Friedman test followed by the Dunnett *post hoc* test. Statistical analysis was performed using the software GraphPad Prism 5 (Prism, San Diego, CA, USA). *p* < 0.05 was considered statistically significant.

## Results

### Characterisation of silver nanoparticles

Table 1 summarizes the physicochemical properties of the AgNSs, and S- and L-AgNWs. Detailed characterisation has been reported previously.^28,30,31^ For all cell exposure experiments, we used DMEM cell culture medium since in our previous report, we showed that this culture medium was not associated with S-AgNW sulphidation or dissolution over time scales as long as 170 hours.^29^ This can be explained by the fact that although Ag^+^ has a high affinity for the sulfur-containing species (cysteine, methionine, and HEPES) present in DMEM, Ag^+^ may not be able to remove sulfur from biological molecules to form an inorganic sulfide without the existence of other oxidizing species.

**Table 1:** Characteristics of silver nanoparticles^a^

Nanoparticles	TEM shape	TEM/SEM (average of 200 particles, nm)	Specific surface area (SSA) for single particle/wire with average diameter	Zeta potential in DI water, pH 7	Free silver ion in the purified product (ppb)
20 nm silver nanospheres	Spherical	14 ± 1.6 nm	40.4 ± 4.8 m^2^ g^−1^	−17.9 ± 3.0 mV	BDL (<1 ppb)
50 nm silver nanospheres	Spherical	49.7 ± 10.5 nm	6.0 ± 0.9 m^2^ g^−1^	− 27.8 ± 0.1 mV	BDL (<1 ppb)
Short silver nanowires	Wire	72 nm (36–108 nm) diameter and 1.5 μm (0.1–3.1 μm) length	4.60 ± 0.2 m^2^ g^−1^	−14.8 ± 0.1 mV	BDL (<1 ppb)
Long silver nanowires	Wire	73 nm (42–128 nm) diameter and 10 μm (0.2–35 μm) length	4.40 ± 0.2 m^2^ g^−1^	−15.2 ± 0.2 mV	BDL (<1 ppb)

a The equation used to measure the specific surface area (SSA) is given in the Appendix using the formula from ref. 43. BDL: below detection limit of ICP-OES; DI: deionised water.

To estimate the conditions under which Ag^+^ may be released from AgNPs, we used inductively coupled plasma optical emission spectroscopy (ICP-OES) to quantify the rates of Ag^+^ ion release from 25 μg mL^−1^ AgNSs and AgNWs (Fig. S1†). To mimic the extracellular and lysosomal pH, the AgNPs were incubated in non-interacting perchlorate buffers with pH 7 and pH 5, respectively, as performed in our previous work.^30,31^ We did not attempt to quantify Ag^+^ release in the DMEM cell culture medium since we have already shown that any Ag^+^ released could bind with complexing anions in the medium, forming insoluble silver compounds (*e.g.* AgCl) and confounding the interpretation of the Ag^+^ release rate *via* ICP-OES.^29^

**Fig. 1:**
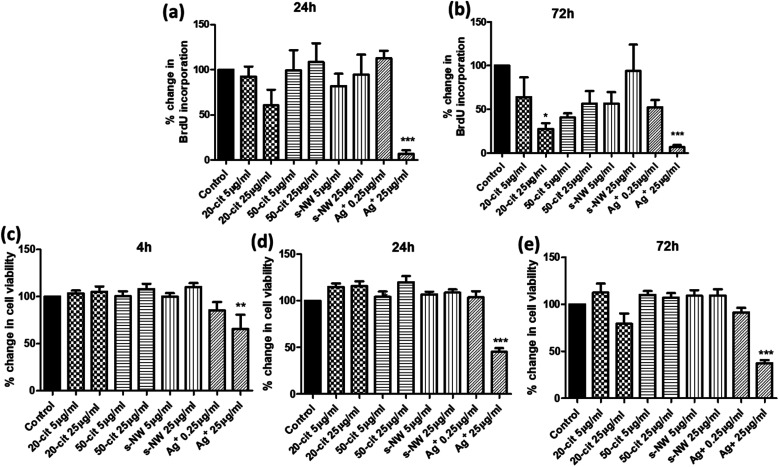
Concentration and time-dependent effect of AgNSs, AgNWs, and Ag^+^ on ASMC proliferation and viability. ASMCs were serum-starved overnight and then incubated with 20 nm or 50 nm AgNSs, S-AgNWs (5 or 25 μg mL^−1^), or Ag^+^ (0.25 or 25 μg mL^−1^). (a and b) Cell proliferation was determined after 24 (a) or 72 h (b) by measuring the rate of DNA synthesis using a BrdU incorporation assay. (c–e) Cell viability was determined after 4 (c), 24 (d) or 72 h (e) using the MTS assay. Bars represent mean ± SEM. Data are expressed as percentage change with respect to the untreated control. Results are representative of mean ± SEM of 5 ASMC donors. *p < 0.05, **p < 0.01 and ***p < 0.001.

We determined the effect of Ag^+^ (0.25 or 25 μg mL^−1^), 20 nm or 50 nm AgNSs (5 or 25 μg mL^−1^), or S-AgNWs (5 or 25 μg mL^−1^) on the rate of DNA synthesis, as a measure of proliferative capacity, and on ASMC viability, at different time points over a 72 h period. Incubation with 20 nm AgNSs (25 μg mL^−1^) led to a time-dependent decrease in the rate of DNA synthesis, which was maximal after 72 h (72.4%; *p* < 0.05; Fig. 1a and b) and was accompanied by a non-statistically significant reduction in cell viability (Fig. 1e). The 50 nm AgNSs and the S-AgNWs did not alter DNA synthesis or viability at any concentration or time point (Fig. 1a–e). Ag^+^ did not have any effect at 0.25 μg mL^−1^, but at 25 μg mL^−1^ it did reduce DNA synthesis (93.3%; *p* < 0.001; Fig. 1a and b) and cell viability (65.1%; *p* < 0.05; Fig. 1c–e) in a time-dependent manner.

**Fig. 2:**
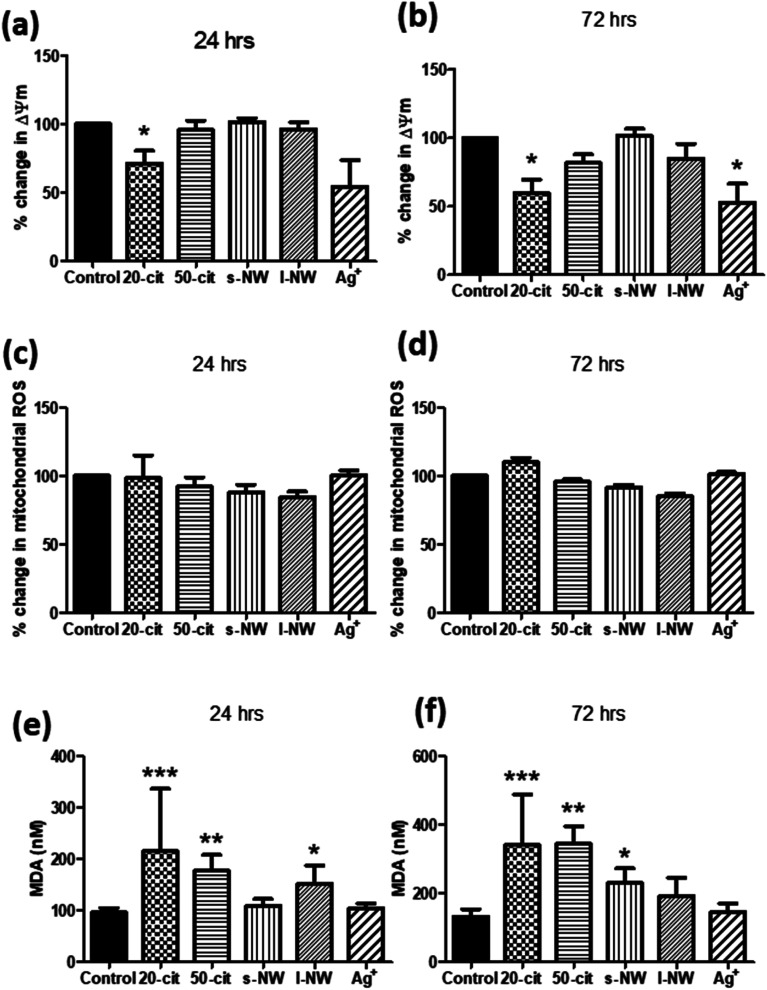
Effect of AgNSs, AgNWs, and Ag^+^ (at the same concentration of silver as present in the particles) on ASMC mitochondrial function and oxidative stress. ASMCs were serum-starved overnight and then incubated with 20 nm or 50 nm nanospheres, S- or L-AgNWs, or Ag^+^ at a concentration of 25 μg mL^−1^ for 24 (a, c and e) or 72 h (b, d and f). Changes in mitochondrial membrane potential (Δ*Ψ*_m_) were determined using the JC-1 assay (a and b) and mitochondrial ROS levels using MitoSOX staining (c and d). Oxidative stress was quantified by measuring the lipid peroxidation product malondialdehyde (MDA; e and f). Bars represent mean ± SEM. Data in panels (a)–(d) are expressed as percentage change with respect to the untreated control. Results are representative of mean ± SEM of 3–5 (a and b), 3 (c and d) or 5 (e and f) ASMC donors. *p < 0.05, **p < 0.01 and ***p < 0.001.

Given the influence of Ag^+^ and 20 nm AgNSs on the proliferation and viability of ASMCs, we investigated the effect of high concentrations of Ag^+^, nanospheres and nanowires on mitochondrial function by determining changes in the mitochondrial membrane potential (Δ*Ψ*_m_). In line with our previous findings, Δ*Ψ*_m_ was reduced by both Ag^+^ (25 μg mL^−1^) and 20 nm AgNSs (25 μg mL^−1^) after 24 and 72 h (Ag^+^: 47.1%, 20 nm: 40.1%; *p* < 0.05), whilst it was not affected by the 50 nm AgNSs (25 μg mL^−1^), and the S- and L-AgNWs (25 μg mL; Fig. 2a and b). The reduction in Δ*Ψ*_m_ in response to Ag^+^ and 20 nm nanospheres was not accompanied by an increase in mitochondrial ROS production (Fig. 2c and d). Nonetheless, pre-treatment of ASMCs with the antioxidant compound *N*-acetyl cysteine (NAC; 10 mM) reversed the reduction in Δ*Ψ*_m_ induced by 20 nm AgNSs (ESI Fig. S2†). This result suggests that the mitochondrial dysfunction induced by the 20 nm AgNSs is ROS-dependent. AgNSs may be a source of oxidative stress as we showed a significant increase in the lipid peroxidation product malondialdehyde (MDA) with both 20 and 50 nm spheres at 24 h (20 nm: 213%; *p* < 0.001, 50 nm: 179.5%; *p* < 0.01) and 72 h post-incubation (20 nm: 259.3%; *p* < 0.001, 50 nm: 260.4%; *p* < 0.01). S- and L-AgNWs also increased MDA, but to a lesser extent, after 24 h (156.2%; *p* < 0.05) and 72 h (170.2%; *p* < 0.05), respectively. Ag^+^ did not modulate MDA levels (Fig. 2e and f).

### Sulfidation of silver nanowires and spheres inside ASMCs

**Fig. 3:**
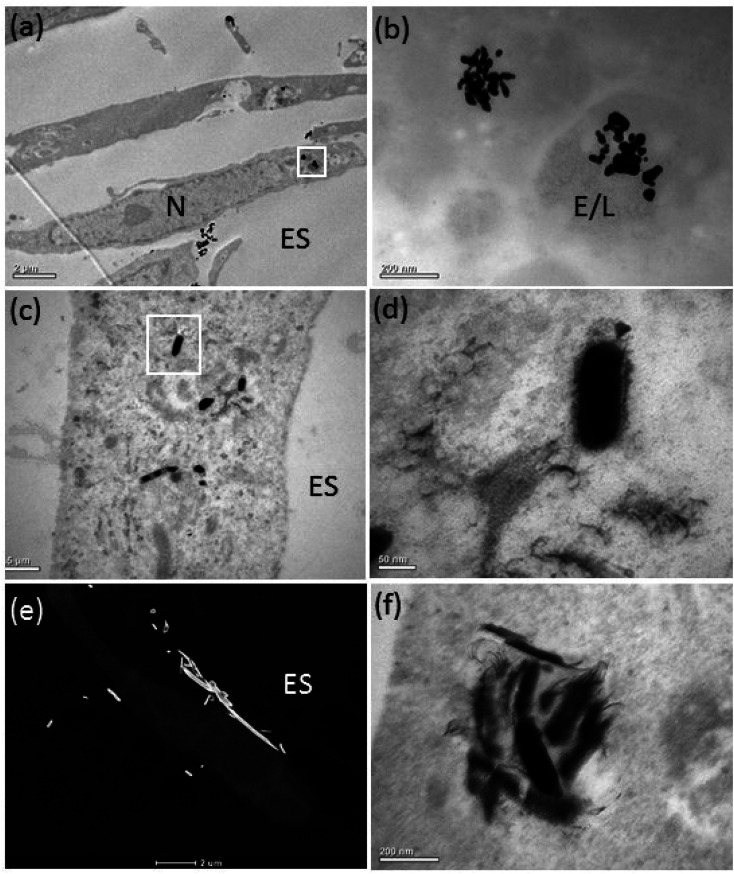
Ag nanostructure dissolution and transformation following uptake by the ASM cells. TEM images showing an altered morphology of 20 nm AgNSs (a and b), 1.2 μm S-AgNWs (c and d) and 10 μm L-AgNWs (e and f) inside ASM cells after 72 h of exposure. Images (b) and (d) are higher magnification images of the nanostructures shown in the boxed regions in (a) and (c). Small diffuse and coalesced particles surrounded the original Ag nanostructures. Figures (a–d) and (f) are bright field TEM images and figure (e) is a HAADF-STEM image. ES = extracellular space; E/L: endosome/lysosome; N = nucleus.

The bright-field (BF) TEM images confirmed ASM uptake of the AgNPs after 72 h of exposure (Fig. 3a–f). Some AgNPs agglomerated inside vesicles (Fig. 3a and b), possibly due to the lower pH of the intravesicular environment and thermodynamic driving force to minimize their total surface energy.^36^ Inside the ASM cells, the morphology of the AgNSs and S- and L-AgNWs was altered and many smaller, diffuse particles surrounded them, indicating dissolution and re-precipitation (Fig. 3b, d and f, respectively). Qualitatively, we observed that the AgNSs and S-AgNWs were avidly internalised by the ASM cells (Fig. 3a, c and d), whereas L-AgNWs were rarely internalised by the cells. Instead these longer wires were occasionally seen at the plasma membrane and outside the cells (Fig. 3e).

**Fig. 4:**
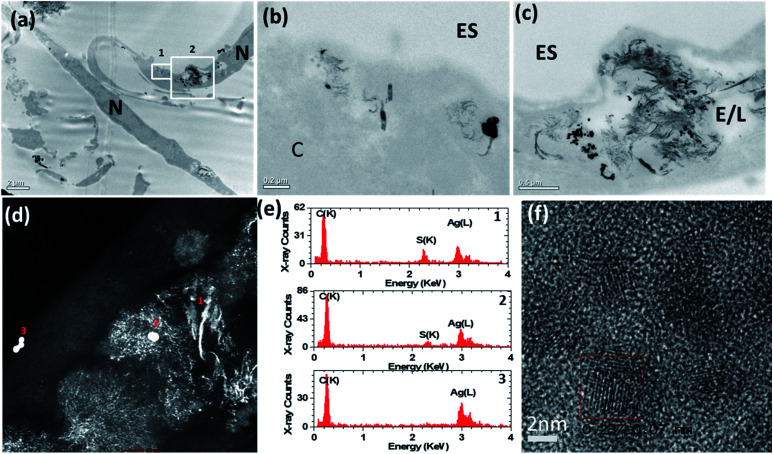
(a)–(c) Bright-field transmission electron microscopy (BF-TEM) images showing the distribution of 20 nm AgNSs inside airway smooth muscle cells after 72 h of incubation with inhibitors of cystathionine β-synthase (CBS) and cystathionine γ-lyase (CSE). A greater number of fine daughter particles surrounded the AgNSs incubated with inhibitors than without inhibitors (Fig. 3) and the spatial distribution of these particles was much more extensive with the inhibitors. (d) HAADF-STEM images also confirmed that many of the AgNSs had dissolved and transformed into smaller diffuse particles. (e) STEM-EDX spectra taken from areas 1–3 marked in (d). (f) High resolution phase contrast image taken from the small diffuse particles. The boxed region shows an individual crystalline particle. ES = extracellular space; E/L: endosome/lysosome; N = nucleus.

To elucidate a mechanism by which the H_2_S-producing enzymes sulfidise the Ag nanomaterials, the 20 nm AgNSs were selected for detailed analysis. In the presence of the CSE and CBS inhibitors, the morphology of the AgNSs was significantly different; a greater number of fine daughter particles surrounded the AgNSs and the size of the AgNSs was smaller, indicating more extensive dissolution than without the inhibitors (Fig. 4a–d). The spatial distribution of these fine particles was also much more extensive in the presence of the inhibitors. The chemical and morphological changes to the intracellular AgNSs under both conditions (with and without the Ag_2_S inhibitors), after 72 h, were analyzed at different positions within the cells exposed to the AgNSs (Fig. 4e). STEM-EDX spectra (Fig. 4e) obtained for the parent AgNSs and fine particles showed Ag(L) peaks and S(K) peaks, indicating association of sulphur on, or around AgNSs (Fig. 4d and e, spectra 2 and 1, respectively). Only NSs near the plasma membrane were composed of pure Ag (spectrum 3). Phase contrast high resolution (HR) TEM images were acquired to characterize the crystalline phase of these fine particles (Fig. 4f). The interplanar spacing of the fine particles was measured from the phase contrast HR-TEM images obtained at the surface and the core of AgNPs. The measured lattice spacings were 0.237 ± 0.007 nm and 0.251 ± 0.007 nm, closely matching the monoclinic structure of Ag_2_S (−103) and (022), respectively (ref. 00-014-0072), supporting the EDX observations.

### Co-localisation of silver nanoparticles with H_2_S producing enzymes

**Fig. 5:**
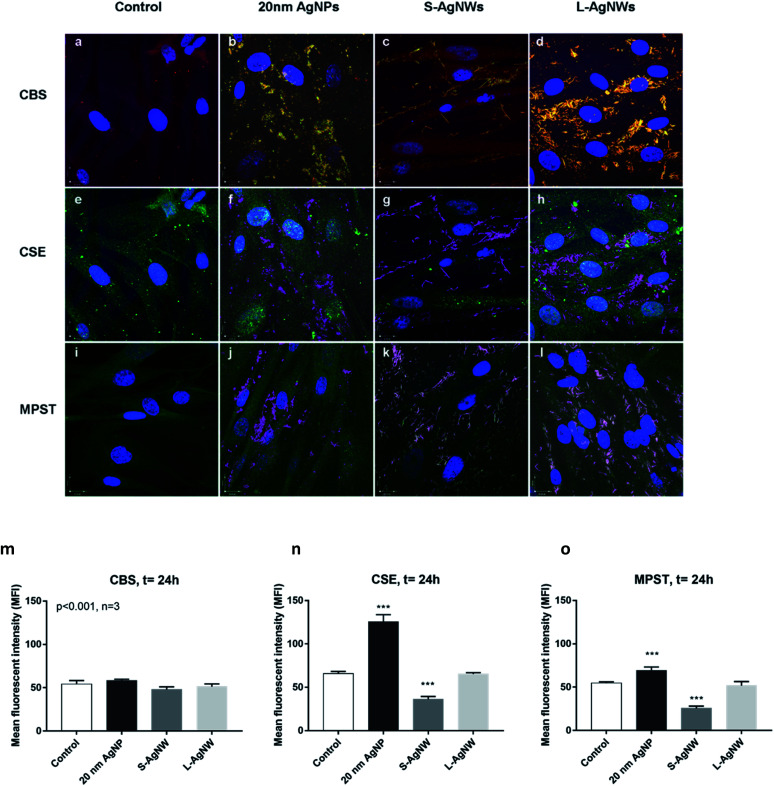
Silver nanomaterial control regulation of specific H_2_S producing enzymes CBS (a–d), CSE (e–h) and MPST (i–l) and the mean fluorescence intensity (MFI) of each enzyme was measured (m–o). Airway smooth muscle (ASM) cells were exposed to silver nanomaterials (AgNSs, S-AgNWs, and L-AgNWs; seen in green (b–d) and magenta (f–h and j–l)) for 24 h and the cells were immuno-stained with fluorescent antibodies against CBS (red), CSE (green) and MPST (green) enzymes and compared with control unexposed cells (a, e and i). Cell nuclei were stained in blue. In some cell regions, the 20 nm AgNSs, S-AgNWs and L-AgNWs co-localised with the CBS (yellow in (b–d)), CSE and MPST (white in (f–h) and (j–l)). The 20 nm AgNSs appeared to upregulate CSE and MPST production (m–o). While the S-AgNWs could significantly downregulate the CSE and MPST (n and o), there was no change of any of the enzymes following exposure to the L-AgNWs (m–o).

Confocal observation revealed the expression of CBS (red), CSE (green) and MPST (green) in ASM cells. The control non-treated cells exhibited a stronger baseline signal of CSE than CBS and MPST suggesting that CSE enzyme could be a major producer of H_2_S in the ASM cell (Fig. 5a, e and i). The localisation of AgNSs and AgNWs was shown using light scattering mode in green (Fig. 5b–d) and magenta (Fig. 5f–h and j–l), respectively. The co-localisation of the enzymes and nanoparticles is shown in yellow (Fig. 5b–d) and white (Fig. 5f–h and j–l) indicating that the H_2_S production may be occurring at the surface of the nanoparticles. The upregulation of H_2_S producing enzymes particularly CSE and MPST was observed in ASM exposed to 20 nm AgNSs following 24 h of exposure (Fig. 5b, f, j and m–o). The presence of S-AgNWs down-regulated the CSE and MPST enzyme expression (Fig. 5m–o), while the L-AgNWs did not affect the regulation of all 3 enzymes (Fig. 5m–o).

## Discussion

AgNPs may directly target primary ASMCs *in vivo* and may be involved in inflammation and in the development of airway hyperresponsiveness observed following pulmonary instillation of these nanoparticles.^9^ In addition, ASMCs play a major role in the pathology of common respiratory diseases, such as asthma and chronic obstructive pulmonary disease, which are characterised by an increase in ASM mass.^23,37^ ASMCs have contractile properties that lead to narrowing of the airways to cause airflow obstruction as observed in these airway diseases to elicit symptoms of wheezing, chest tightness and breathlessness, in addition to contributing to the inflammatory process.^22,38^ Indeed, we have shown that instillation of AgNPs in rats can induce pulmonary eosinophilic and neutrophilic inflammation with induction of bronchoconstriction and bronchial hyperresponsiveness, features that are characteristic of asthma.^9^ However, little is known about the direct effects of nanoparticles on the human ASM cell. Thus, our study is one of the first to determine the effect and fate of AgNPs on the human ASM cell.

Our experiments have shown that only the Ag^+^ control solution, at the concentration that would be delivered inside the cells if the particle dissolved fully, reduced ASM cell proliferation and viability to a very large extent. The S-AgNWs did not affect proliferation or cell death at the maximal concentration of 25 μg mL^−1^, while both the S- and L-AgNWs also did not affect mitochondrial membrane potential and mitochondrial reactive oxygen species (ROS) production, but only caused a small release of malondialdehyde from the ASM cells that indicated the release of total ROS. However, the MDA release, associated with greater total ROS, was observed for all the AgNSs but only the 20 nm AgNSs caused a significant reduction in mitochondrial membrane potential. These dependencies of particle size and geometry relate to differences in Ag^+^ release rates associated with changes in the diameter and especially the specific surface area for the nanoparticles (provided in Table 1) that are avidly internalised by the ASM cells^21^ (ESI Fig. S1†), even though the surface chemistry of the wires and spheres is different. In contrast, the soluble Ag^+^ concentration (1% AgNO_3_ of 25 μg mL^−1^) equivalent to a concentration of ionic silver which was likely to dissolve from the particles during the exposure time caused cell death and a significant reduction in membrane potential without affecting mitochondrial ROS or release of MDA, which indicates that high local intracellular dose of Ag^+^ delivered in close proximity to the mitochondria by the AgNSs is likely to be the cause of mitochondrial damage. Thus, the concentration of silver ions representative of dissolving silver ions and the 20 nm AgNSs were the most toxic.

The addition of a thiol scavenger and antioxidant, *N*-acetylcysteine (NAC), significantly prevented the toxicity of 20 nm AgNSs and Ag^+^, suggesting that dissolution to silver ions was a mechanism for the toxicity. Moreover, we investigated the roles of endogenous H_2_S and the main H_2_S producing enzymes in the process of sulfidation, which is thought to ‘trap’ silver ions and thus reduce their toxicity to cells.^21^ We showed that intracellular inhibition of the H_2_S-producing enzymes increased dissolution of the 20 nm AgNSs, which we speculated was due to a lower amount of H_2_S present in the cells to mop up the Ag^+^ that dissolved from the particle surface. In other work,^21^ we have shown no traces of sulfidation on the surface of PVP-capped AgNWs incubated with cysteine alone or in DMEM cell culture medium, which contains the sulfur-containing components cysteine, methionine, and HEPES (2-[4-(2 hydroxyethyl)piperazin-1-yl]-ethanesulfonic acid, a buffering agent), indicating that sulfate- and sulfur-containing amino acids do not lead to sulfidation. This finding supports the notion that H_2_S contributes to Ag sulfidation and suggests that Ag^+^ may not be able to remove sulfur from biological molecules to form an inorganic sulfide without the existence of other oxidizing species.

A lack of standardisation of cellular methods for assessing the toxicity and the physiochemical character of nanoparticles for nanotoxicological studies is likely to lead to erroneous and highly variable experimental results. This variability is especially manifested for silver nanoparticles, where it is still not understood whether toxicity in mammalian cells is driven by the physical particle or by dissolved ions or a combination of both, due to inconsistent data between studies.^21,39^ The results of these studies may have been confounded by not taking into account a thermodynamically favoured reaction of silver with free sulfide species inside the cells, to form highly insoluble Ag_2_S species.^21^ Ag_2_S has been shown to have lower dissolution rates and induces lower toxicity than non-sulfided silver species in mammalian cells.^21^ Moreover, surface ‘sulfidation’ reactions may also occur in some cell culture media, independent of any biological effects, depending on their constituents, or even during particle storage due to adventitious air pollutants.^21,29^ It is, therefore, essential to confirm that Ag particle surfaces are not sulfided prior to incubation with the cells, if the intrinsic toxicity of the silver species is to be determined.^21^ We characterised the extent of sulfidation and rate of dissolution of our in-house silver nanoparticles, both in water and in the ‘exposure medium’ so that these parameters are known at the point of contact with the cells and valid conclusions can be drawn about the physicochemical characteristics linked with bio-reactivity.^21^

In addition to standard characterisation of size, zeta potential and agglomeration, we also previously modelled dissolution at different pHs and with constituents of lung lining fluid to account for altered agglomeration states and dissolution rates which may occur following immersion with a pulmonary surfactant or *in vivo* uptake into cells in the peripheral lung. For the particles used in this study, we found that dissolution of the 20 nm AgNSs in de-ionised water and in dipalmitoylphosphatidylcholine (DPPC), the main phospholipid constituent of pulmonary surfactants, was negligible for up to 1 week.^28^ The solubility of AgNWs was also negligible when incubated with de-ionised water, buffer solutions containing bovine serum albumin or different combinations of pulmonary surfactant constituents.^31^ However, the dissolution was shown to be pH-sensitive, with the rate increasing at pH 5,^28,31^ for all Ag nanostructures; the relative rates of dissolution decreased systematically with diameter: 20 nm AgNSs > 50 nm AgNSs > S-AgNWs ≈ L-AgNWs (ESI Fig. S1† – note that the rates do not change between 72 and 24 hours for the AgNS and AgNWs^29,40^). These *ex situ* findings suggest that the rate of dissolution for the 20 nm particles may be substantially higher if taken inside compartments of the cell such as the lysosome (pH 5). Importantly, we also confirmed that the Dulbecco's modified Eagle's medium (DMEM), used for cell treatments, is not associated with silver sulfidation during the time-course of our experiments.^21^

In previous work, our group showed that incubation of AgNPs in perchlorate buffer (pH 5, corresponding to the average pH of endosomes) led to an increase in free Ag^+^ in solution at both 4 h and 24 h.^28^ Similarly, in the current study, extensive dissolution of the AgNPs was observed directly, inside the lysosomes, after cell uptake. Interestingly, the amount of dissolution of the 20 nm AgNSs and spatial coverage of the fine particulate Ag_2_S material was significantly greater in the presence of the CBS and CSE inhibitors. These inhibitors are expected to reduce the amount of H_2_S available to react with the Ag^+^ which dissolves from the AgNPs; therefore, the Ag^+^ diffuses further and for a longer time. With a lower background H_2_S concentration, the area of depletion around the parent AgNPs, due to sulphidation reactions, will be larger, and thus the ions will gradually travel further to reach a sufficient concentration of H_2_S to form Ag_2_S. The stark difference in the amount of dissolution and transformation of the AgNPs in the presence and absence of the inhibitors, further strengthens our hypothesis that the H_2_S produced by CSE and CBS (and also PMST) results in intracellular sulfidation of the silver nanoparticles in human cells.

The immunohistochemical studies of the 3 enzymes, CBS, CSE and PMST, underlying the production of H_2_S provided further insight into the interaction of these enzymes with AgNPs inside the ASM cell. First, this shows that H_2_S production may be occurring at the surface of the AgNPs, which would imply that the process of sulfidation would very much depend on the surface area of the particle, favouring very much the smaller sized AgNSs rather than the AgNWs. While the S-AgNWs and 20 nm AgNSs co-localised with all 3 H_2_S enzymes, only the 20 nm AgNSs augmented CSE and MPST protein expression while the S-AgNWs reduced CSE expression and the L-AgNWs had no effect. The implication of this observation is that AgNSs would be more rapidly sulfided than the NWs, because they have a larger specific surface area of dissolution. The mechanism by which that AgNPs modulate the expression of these H_2_S enzymes is unclear but it would be interesting to speculate whether this could be a direct effect of silver ions.

There are weaknesses that need to be addressed: 2 different types of coatings NSs (citrate-coated) *vs.* NWs (long axis of the wires coated with PVP) were used and the amount of uptake of the different AgNSs was not quantified. The reason for using two different coating agents was because, on the one hand, there is no established synthesis method for the high-yield fabrication of citrate-coated AgNWs with well-controlled sizes and, on the other hand, the synthesis of PVP-coated AgNPs involves complicated preparation procedures, with difficulties in attaining pure and monodisperse *spherical* particles. It is well established that a PVP coating will reduce the toxicity of the AgNWs compared to citrated coated AgNWs. A study found that (PVP)-AgNPs were less toxic than citrate-AgNPs with the same diameter as citrate-AgNPs.^41^ To our knowledge, there is no direct evidence showing whether or how a citrate coating will impact on the sulfidation process of AgNPs. Indeed, citrate capped on AgNP surfaces through carboxyl acid groups interacting with Ag can provide electrostatic repulsive forces to protect AgNPs against aggregation. It is reasonable to expect that the nature of the organic coating may affect the kinetics of AgNP sulfidation by altering the rate at which the S-containing species, *e.g.* H_2_S, HS^−^, S_2_^−^ penetrate through the organic layer, to reach the Ag nanoparticle surface and react with Ag^+^ and form silver sulfide. In our previous work, we showed that the rate and amount of dissolution were lower for PVP capped 20 nm AgNPs than citrate coated 20 nm AgNPs over 72 hours.^40^ However, the capping agent is unlikely to significantly alter the Ag sulfidation process itself, as Ag has a much higher affinity to sulfur than oxygen, and the formation of highly stable silver sulfide is thermodynamically favoured (Tables 2 and 3).

**Table 2:** Stability constants (log *K*_f_) for Ag^+^ – organic compounds showing that silver binds much more strongly to sulfur than oxygen groups^44^

Compound	Formula	Log *K*_f_
Phenol	C_6_H_5_OH	0.34
Acetic acid	CH_3_COOH	0.73
Methylamine	CH_3_NH_2_	3.06
Dimethyl sulphide	CH_3_NH_2_	3.7
Cysteine	–OOCCH(NH_3_^+^)CH_2_SH	11.9

**Table 3:** Solubility products (*K*_sp_) of silver compounds showing that silver sulfide has much lower solubility than other silver compounds^44^

Compound	Formula	*K*_sp_
Silver oxide	Ag_2_O	4.00 × 10^−11^
Silver chloride	AgCl	1.77 × 10^−10^
Silver sulphide	Ag_2_S	5.92 × 10^−51^

Nevertheless, we still observed strong relationships between the geometric size of the AgNS and altered markers of cell health. These results, together with the data shown in ESI Fig. S1,† comparing Ag ion release for each NS type, demonstrate that the toxicity of AgNSs is correlated with the amount of bioavailable Ag^+^. Images showing cellular uptake of the 50 nm AgNS were also missing; however, it is expected that the 50 nm AgNS would also be internalised by the ASMCs as the smaller 20 nm and larger 1.5 μm long AgNWs are internalised. We note, however, that although Ag^+^ release is a major pathway for the biological activity of nanosilver, AgNP toxicity can also be attributed to particle surface reactions,^42^ which generate ROS or catalyze the oxidation of cellular components.

In summary, we show that the intracellular toxicity of these AgNPs in ASM cells in relation to cell proliferation and death, mitochondrial membrane potential and ROS production, was determined by the degree of solubility of Ag^+^ released and the degree of the sulfidation process, effects that were related to the particle size and geometry. These 2 effects can be considered to be antagonistic in terms of the toxicity outcome because the sulfidation process would inactivate the effect of Ag^+^.^21^ The observation that the greatest toxicity was found with the 20 nM AgNS, and not with the AgNWs, illustrates the fact that the degree of Ag^+^ release from the AgNS would not be sufficiently counteracted by the sulfidation process. These *in vitro* data are in fact concordant with the *in vivo* measurements reflecting ASM physiology obtained in rats instilled with AgNPs with different diameters. Thus, both the 20 and 110 nm AgNSs caused bronchoconstriction while only the 20 nm AgNSs caused bronchial hyperresponsiveness,^10^ while the L- and S-AgNWs did not induce bronchoconstriction with the L-AgNWs causing a mild degree of bronchial hyperresponsiveness.^26^

## Appendix

### Equation used to measure the specific surface areas of silver nanoparticles. From Davey 1925

The specific surface area of the Ag nanospheres and wires is given by eqn (1) and (2) below.1
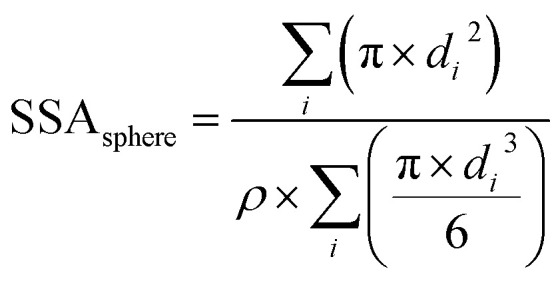
2
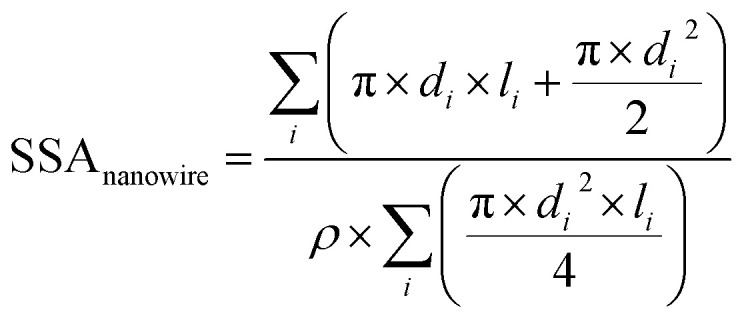


For the nanowires, *l* is the length of the cylinder and *d* is the diameter of the base of the cylinder (assuming the cylinder is flat). In eqn (1), *i* refers to each individual particle in the TEM images, *d*_*i*_ is the diameter of particle *i*, and ρ is the density of metallic silver (10.50 g mL^−1^).^43^

## Conflicts of interest

There are no conflicts to declare.

## Supplementary Material

NA-002-D0NA00745E-s001
