# Supplementary material for: Effect of silver nanospheres and nanowires on human airway smooth muscle cells: role of sulfidation
Source: Nanoscale Adv. 2020 Oct 9;2(12):5635–47. doi: 10.1039/d0na00745e (PMC8330518; doi:10.1039/d0na00745e)
Supplement: NA-002-D0NA00745E-s001 [file NA-002-D0NA00745E-s001.pdf]

Electronic Supplementary Material (ESI) for Nanoscale Advances.
This journal is © The Royal Society of Chemistry 2020

Supplementary figures

Effect of silver nanospheres and nanowires on human airway smooth muscle cells *in vitro*: role of sulfidation

Charalambos Michaeloudes[1], Joanna Seiffert[1], Shu Chen[2], Pakatip Ruenraroengsak[2,3], Leo Bey[2,4], Ioannis G. Theodorou[2], Mary Ryan[2], Xiaoxing Cui[5], Jim Zhang[5], Milo Shaffer[2], Terry Tetley[1], Alexandra E. Porter[2], Kian Fan Chung[1]

[1]National Heart & Lung Institute, Imperial College, London SW3, UK; [2]Department of Materials and London Centre for Nanotechnology, Imperial College, London SW3, UK; [3] Faculty of Pharmacy, Mahidol University, Bangkok, Thailand, 10400; [4]Faculty of Medicine, University of Malaya, Kuala Lumpur 50603, MYS; [5]Nicholas School of Environment & Duke Global Health Institute, Duke University, Durham, USA

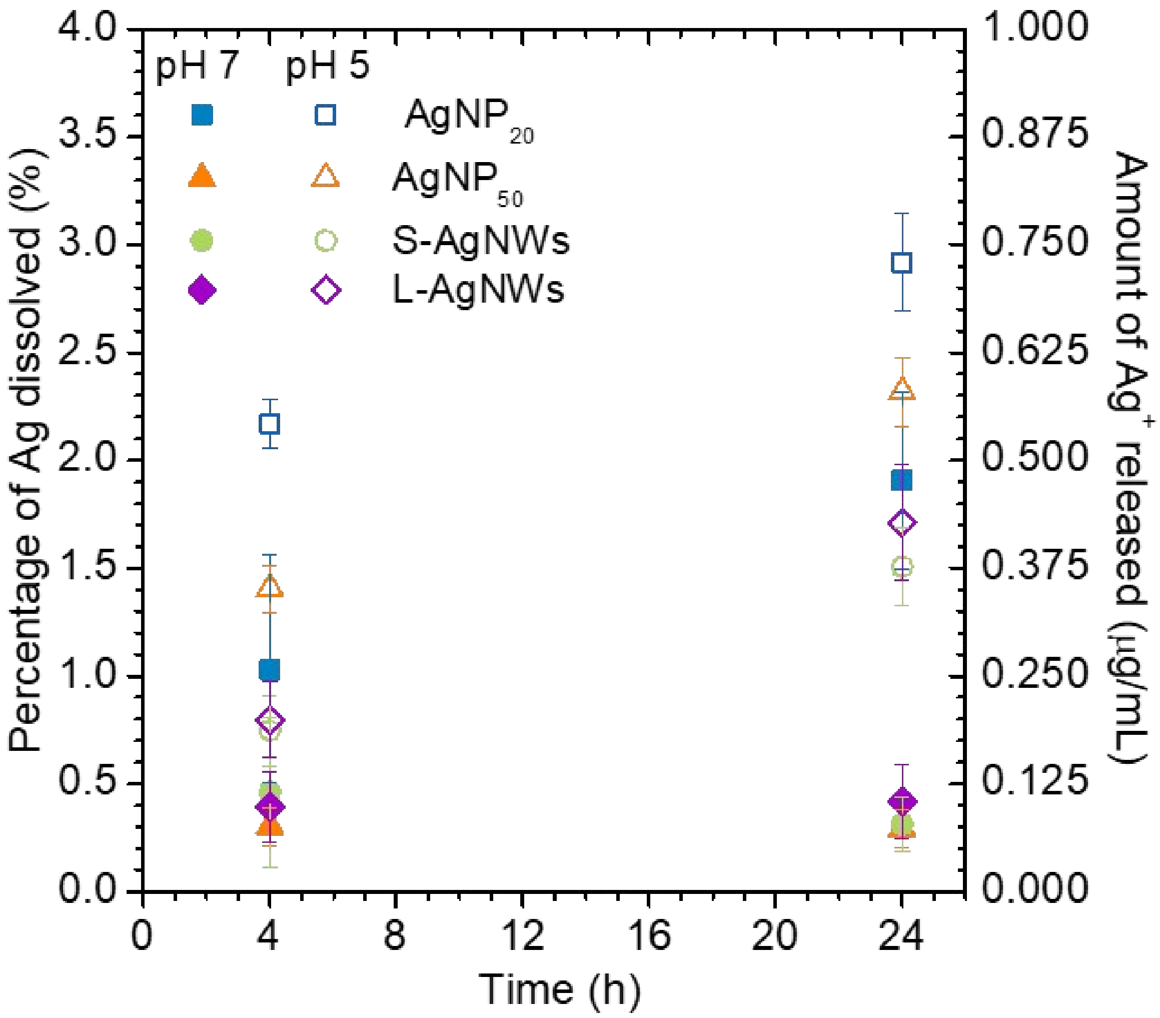

Supplementary Figure 1

ICP-OES analysis of the percentage and amount of free Ag^+ ions released from $AgNP_{20}$, $AgNP_{50}$, S-AgNWs, and the L-AgNWs, incubated in perchlorate buffer solutions with pH 7 (solid symbols) and pH 5 (open symbols), for 4 and 24 h . Adapted from (1, 2).

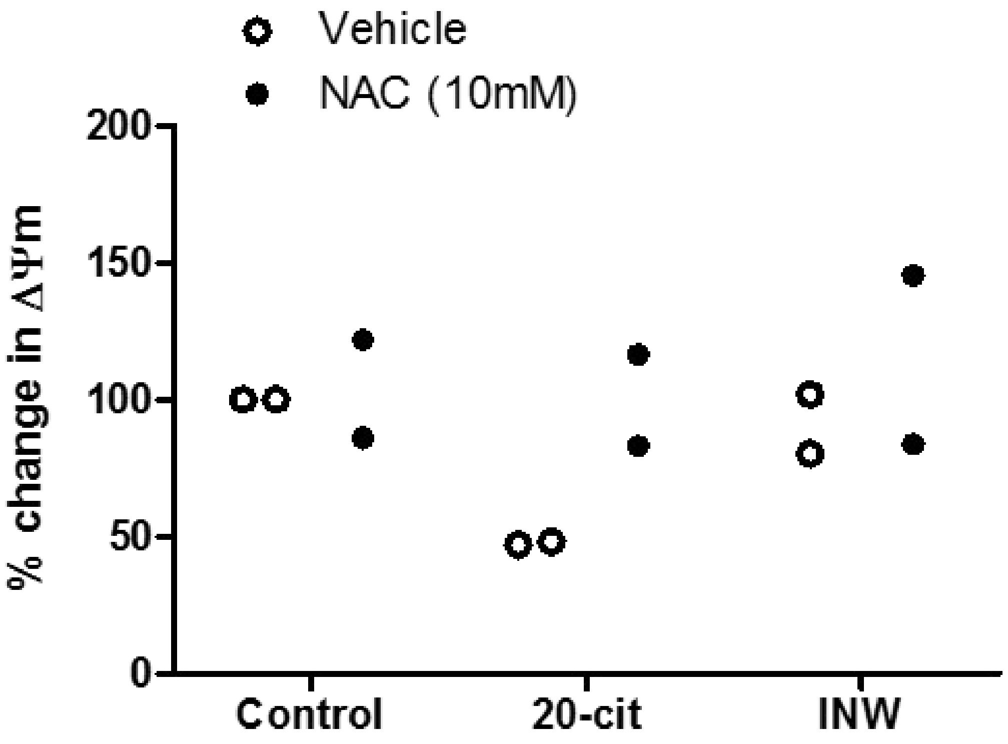

Supplementary Figure 2.

Effect of N-acetylcysteine on silver nanosphere- and nanowire-dependent mitochondrial membrane potential. ASMCs were serum-starved overnight, pre-treated with N-acetyl cysteine (NAC; 10mM) or vehicle for 1hr and then incubated with 20µm nanospheres (25 µg/mL) or long nanowires (lNW; 25 µg/mL) for 72hrs. Changes in mitochondrial membrane potential (ΔΨm) were determined using the JC-1 assay. Each data point represents results from one ASMC donor.

References

1. Theodorou IG MK, Chen S, Goode AE, Yufit V, Ryan MP, Porter AE. . Silver Nanowire Particle Reactivity with Human Monocyte-Derived Macrophage Cells: Intracellular Availability of Silver Governs Their Cytotoxicity. ACS BIOMATERIALS SCIENCE & ENGINEERING 3(10): 2017 :2336-47.

2. Leo BF, Chen S, Kyo Y, Herpoldt KL, Terrill NJ, Dunlop IE, et al. The stability of silver nanoparticles in a model of pulmonary surfactant. Environmental science & technology. 2013;47(19):11232-40.
